# A pharmacoeconomic evaluation of aprepitant injection versus aprepitant capsules for the prevention of CINV caused by moderately and highly emetogenic chemotherapy 

**DOI:** 10.3389/fphar.2026.1879202

**Published:** 2026-08-04

**Authors:** Zhen Chen, Ming Cai, Yanyan Dong, Liang Jin, Mingzhen Li, Lei Shen, Xuan Jiang

**Affiliations:** 1 Department of Pharmacy, The Third People’s Hospital of Hefei, Hefei, Anhui, China; 2 Department of Pharmacy, The Second Affiliated Hospital of Anhui University of Chinese Medicine, Hefei, Anhui, China; 3 Department of Oncology, The Third People’s Hospital of Hefei, Hefei, China

**Keywords:** chemotherapy-induced nausea and vomiting, aprepitant injection, aprepitant capsules, pharmacoeconomics, cost-effectiveness analysis, propensity score matching

## Abstract

**Objective:**

To compare the clinical efficacy, safety, and pharmacoeconomic value of aprepitant injection versus aprepitant capsules in preventing chemotherapy-induced nausea and vomiting (CINV) associated with moderately and highly emetogenic chemotherapy, and to provide evidence for optimizing antiemetic strategies and rational healthcare resource allocation.

**Methods:**

This real-world observational study retrospectively included patients with solid tumors who received moderately to highly emetogenic chemotherapy regimens in our hospital from January 2023 to December 2025. Among 368 eligible patients, 62 received aprepitant capsules and 306 received aprepitant injection as part of a. Standard triple antiemetic regimen (NK- one receptor antagonist + 5-HT3 receptor antagonist + dexamethasone). After 1:1 propensity score matching (PSM), 60 matched pairs were included for comparative analysis. Clinical efficacy outcomes included complete response (CR), no nausea rate (NCR), no vomiting rate (NVR), and no rescue treatment rate (NRR) during the overall (0–120 h), acute (0–24 h), and delayed (24–120 h) phases. Quality of life was assessed using the EORTC QLQ-C30 questionnaire. Safety indicators included treatment-related adverse events. Pharmacoeconomic evaluation was conducted from the healthcare system perspective, incorporating antiemetic drug costs, total medication costs (antiemetics, chemotherapy drugs, adjuvant drugs), examination fees, laboratory costs, treatment fees, and total hospitalization costs. Cost-effectiveness analysis (CEA), cost-utility analysis (CUA), incremental cost-effectiveness ratio (ICER), incremental cost-utility ratio (ICUR), Monte Carlo simulation, and cost-effectiveness acceptability curves (CEAC) were applied.

**Results:**

After PSM, baseline characteristics between groups were well balanced (P > 0.05). The overall complete response rates were 78.3% in the injection group and 76.7% in the capsule group, with no statistically significant difference (P > 0.05). Acute and delayed phase CR rates, NCR, NVR, and NRR were also comparable between groups (all P > 0.05). Both groups showed significant post-chemotherapy improvements in EORTC QLQ-C30 functional and global health scores, with reduced symptom burden (P < 0.05), while intergroup differences remained non-significant (P > 0.05). The average antiemetic drug cost was significantly lower in the injection group than in the capsule group due to lower acquisition costs. The mean total hospitalization cost was lower in the injection group than in the capsule group (12,856.42 vs. 13,968.57 RMB), corresponding to an incremental cost difference of −1,112.15 RMB. Cost-effectiveness analysis yielded an incremental cost-effectiveness ratio of −66,996.99 RMB per additional complete response achieved, indicating lower costs with slightly improved effectiveness. Cost-utility analysis yielded an incremental cost-utility ratio of −111,215.00 RMB per unit improvement in global health status score. Monte Carlo simulation and CEAC confirmed the robustness of the pharmacoeconomic advantage of aprepitant injection across a wide range of willingness-to-pay thresholds. Safety profiles were comparable, with no significant differences in adverse event incidence.

**Conclusion:**

Aprepitant injection and aprepitant capsules showed no statistically significant differences in efficacy, safety, or quality-of-life outcomes in this matched retrospective cohort. However, aprepitant injection demonstrated superior pharmacoeconomic performance through lower antiemetic drug costs, reduced total hospitalization expenditures, and more favorable economic outcomes. From a healthcare resource utilization perspective, aprepitant injection may represent a more cost-effective prophylactic antiemetic strategy and could provide useful evidence for individualized clinical decision-making and reimbursement policy optimization.

## 1 Introduction

Malignant tumors remain one of the leading global public health challenges and continue to impose substantial disease burden worldwide. With ongoing population aging, environmental changes, and lifestyle transitions, both the incidence and mortality of cancer have shown persistent upward trends in recent years (Sung et al., 2021). Chemotherapy remains an essential component of comprehensive antitumor treatment for many solid malignancies. However, despite its therapeutic efficacy, chemotherapy is frequently accompanied by a variety of adverse reactions, among which chemotherapy-induced nausea and vomiting (CINV) is one of the most common and distressing complications (Gupta et al., 2021). Previous studies have reported that approximately 70% −80% of patients receiving chemotherapy experience varying degrees of nausea or vomiting, particularly when moderately or highly emetogenic chemotherapy regimens are administered (Lorusso et al., 2020). Without standardized prophylactic antiemetic intervention, the incidence of CINV may exceed 90% in highly emetogenic chemotherapy and may range from 30% to 90% in moderately emetogenic regimens (Chow et al., 2023). CINV not only causes severe physical discomfort, but may also contribute to dehydration, electrolyte disturbances, nutritional deterioration, reduced functional status, and substantial impairment in quality of life (Xie et al., 2021). More importantly, poorly controlled CINV can reduce patient adherence to chemotherapy, potentially leading to delayed treatment cycles, dose reductions, or premature discontinuation, thereby negatively affecting long-term antitumor efficacy (Bos et al., 2020). Therefore, optimizing CINV prevention is of major importance for both supportive cancer care and treatment continuity.

As the pathophysiological understanding of CINV has evolved, antiemetic strategies have become increasingly standardized. Current antiemetic regimens primarily include 5-hydroxytryptamine-3 (5-HT3) receptor antagonists, neurokinin-1 (NK- 1) receptor antagonists, glucocorticoids, and other supportive agents. Among these, NK- one receptor antagonists play a particularly important role by selectively blocking substance P-mediated emetic signaling pathways and have demonstrated strong efficacy in controlling both acute and delayed CINV (Dranitsaris et al., 2022). International clinical guidelines currently recommend triple antiemetic prophylaxis consisting of an NK- one receptor antagonist, a 5-HT3 receptor antagonist, and dexamethasone for patients receiving moderately to highly emetogenic chemotherapy (Bosnjak et al., 2025; Abe et al., 2023). Aprepitant, a widely used NK- one receptor antagonist, has demonstrated favorable efficacy and safety profiles in clinical practice. It is currently available in both oral capsule and intravenous injection formulations (Authors and Severn, 2022). In standard clinical application, aprepitant capsules are administered orally at 125 mg approximately 1 h before chemotherapy on day 1, followed by 80 mg orally each morning on days 2 and 3, whereas aprepitant injection is typically administered intravenously at 130 mg approximately 30 min before chemotherapy as a single-dose regimen. Compared with oral administration, intravenous formulations may provide practical advantages in ensuring medication adherence, reducing administration complexity, and improving treatment convenience, especially in patients with severe gastrointestinal symptoms, poor oral intake, or swallowing difficulties (Wang et al., 2024). In addition, significant differences in acquisition costs and administration patterns between formulations may lead to varying healthcare economic outcomes.

With rising healthcare expenditures and increasing emphasis on value-based medicine, pharmacoeconomic evaluation has become an increasingly important component of clinical decision-making and reimbursement policy development. Pharmacoeconomic analysis systematically compares treatment costs with clinical outcomes, thereby providing evidence for optimizing resource allocation and improving healthcare efficiency (Boscolo Bielo et al., 2023; Chen et al., 2021). Although aprepitant has been widely incorporated into antiemetic protocols, most existing studies have focused primarily on efficacy and safety, while direct pharmacoeconomic comparisons between intravenous and oral formulations remain limited. In particular, real-world evidence regarding the comparative cost-effectiveness of aprepitant injection versus aprepitant capsules in patients receiving moderately and highly emetogenic chemotherapy remains insufficient.

Therefore, this real-world observational study was conducted to compare the clinical efficacy, quality-of-life impact, safety, and pharmacoeconomic outcomes of aprepitant injection and aprepitant capsules in solid tumor patients undergoing moderately to highly emetogenic chemotherapy. By integrating propensity score matching (PSM), cost-effectiveness analysis (CEA), and cost-utility analysis (CUA), this study aimed to provide more comprehensive evidence regarding the economic and clinical value of different aprepitant formulations, thereby offering practical support for individualized antiemetic strategy selection and healthcare reimbursement optimization.

## 2 Materials and methods

### 2.1 Study design and participants

**FIGURE 1:**
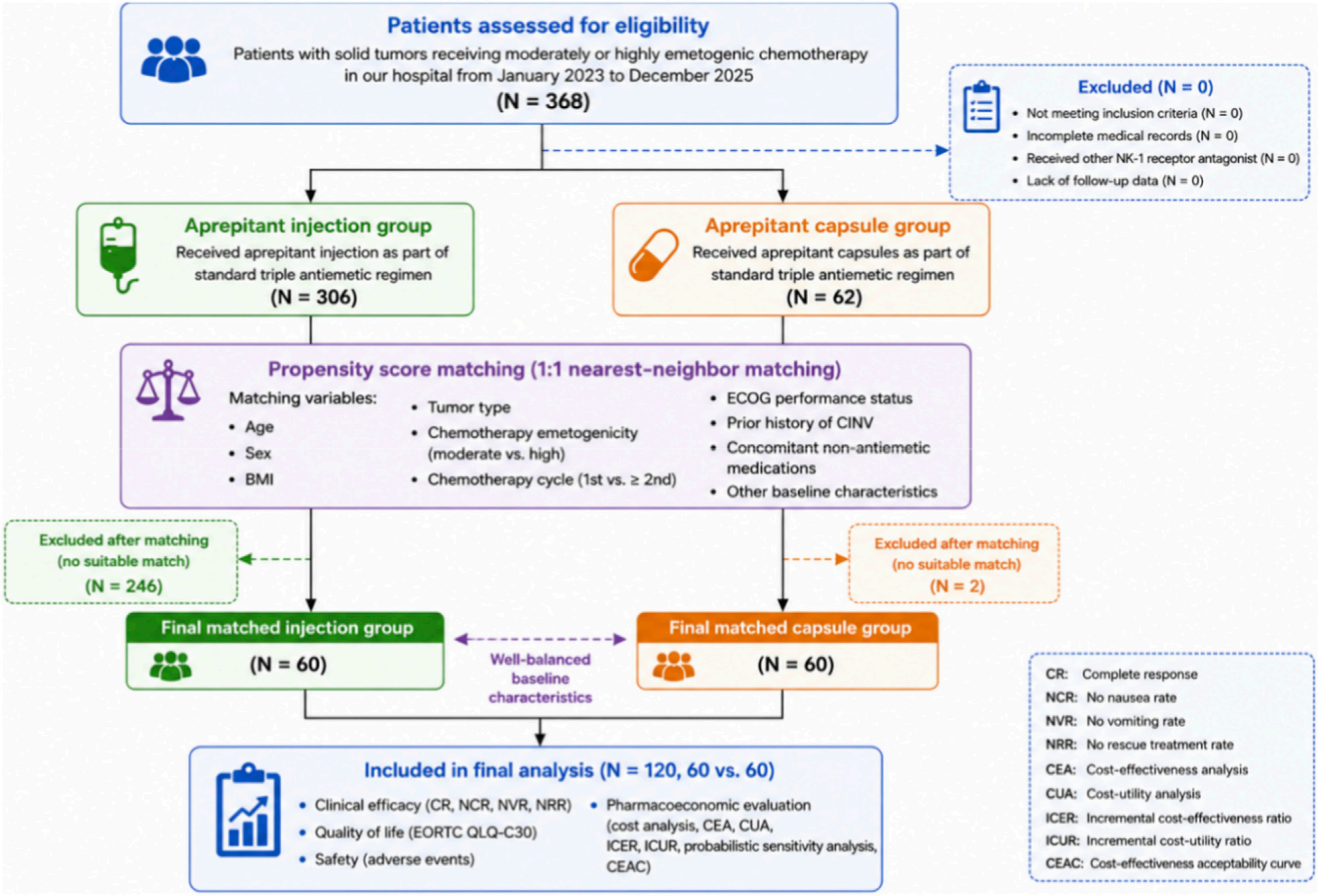
Study flow diagram of patient selection and propensity score matching.

This single-center real-world retrospective observational study was conducted at the Department of Oncology, The Third People’s Hospital of Hefei, a tertiary general hospital located in Hefei, Anhui Province, China. The study retrospectively compared the clinical efficacy, safety, and pharmacoeconomic value of aprepitant injection versus aprepitant capsules for the prevention of chemotherapy-induced nausea and vomiting (CINV) in adult patients with solid tumors receiving moderately or highly emetogenic chemotherapy between January 2023 and December 2025. Clinical information, antiemetic treatment records, follow-up outcomes, and hospitalization cost data were collected from the hospital electronic medical record system. All patient data were anonymized to ensure patient privacy and data security. Clinical information, treatment records, medical expenditure data, and follow-up information were collected from the hospital electronic medical record system and related clinical follow-up databases. Patients with histologically confirmed solid tumors who received moderately or highly emetogenic chemotherapy regimens in our hospital between January 2023 and December 2025 were consecutively screened. During the study period, a total of 368 eligible patients met preliminary inclusion criteria, among whom 306 patients received aprepitant injection and 62 patients received aprepitant capsules as part of standard triple antiemetic prophylaxis consisting of an NK- one receptor antagonist, a 5-hydroxytryptamine-3 (5-HT3) receptor antagonist, and dexamethasone. Given the substantial baseline imbalance in group size and potential confounding factors in real-world treatment allocation, propensity score matching (PSM) was performed to improve intergroup comparability and reduce selection bias. Matching variables included demographic and clinical characteristics that could potentially influence antiemetic efficacy, such as age, sex, tumor type, chemotherapy emetogenicity, chemotherapy cycle, and concomitant non-antiemetic medications used for underlying disease management. A 1:1 nearest-neighbor matching method with an appropriate caliper width was applied. After matching, 60 matched pairs (60 patients in the injection group and 60 patients in the capsule group) were successfully included in the final comparative analysis. Both groups received guideline-recommended triple antiemetic prophylaxis throughout the chemotherapy cycle. Clinical efficacy, quality of life, safety outcomes, and pharmacoeconomic indicators were subsequently analyzed based on the matched cohort. The study protocol was reviewed and approved by the Ethics Committee of The Third People’s Hospital of Hefei (Ethical approval number: LC24YPJJBJ01). Because this was a retrospective observational study using anonymized medical records and follow-up data, the requirement for written informed consent was waived by the Ethics Committee. No additional intervention was performed, and no additional biological specimens were collected specifically for this study. All data were processed confidentially in accordance with the Declaration of Helsinki. The overall patient screening and matching process is presented in Figure 1.

### 2.2 Inclusion and exclusion criteria

(1) Inclusion criteria: ① Patients with solid tumors confirmed by histopathological diagnosis; ② Patients receiving moderately or highly emetogenic chemotherapy regimens; ③ Patients using aprepitant injection or aprepitant capsules as the basic antiemetic drug combined with a 5-HT3 receptor antagonist and dexamethasone for prophylactic antiemetic therapy; ④ Age ≥18 years; ⑤ Complete clinical data with the ability to complete follow-up and relevant indicator recording.

(2) Exclusion criteria: ① Patients with a history of allergy to aprepitant or its excipients; ② Pregnant or lactating women; ③ Patients with severe hepatic or renal insufficiency or severe mental illness; ④ Patients who had participated in other drug clinical trials within the past 3 months; ⑤ Patients with incomplete clinical data or unable to complete follow-up during the study period.

### 2.3 Treatment regimens

(1) Injection group: Patients received an intravenous infusion of aprepitant injection 130 mg approximately 30 min before the start of chemotherapy, combined with a 5-HT3 receptor antagonist (ondansetron 16–24 mg) and dexamethasone 6 mg. Chemotherapy was then administered according to the planned regimen. The subsequent administration of dexamethasone was determined according to the emetogenic risk of the chemotherapy drugs and clinical requirements. (2) Capsule group: Patients orally received aprepitant capsules 125 mg approximately 1 h before chemotherapy, combined with a 5-HT3 receptor antagonist and dexamethasone. Aprepitant capsules 80 mg were then taken orally once daily on days 2 and 3. Other treatments were consistent with those in the injection group. No additional antiemetic agents with independent antiemetic activity, including olanzapine or supplementary dexamethasone beyond the standard prophylactic regimen, were routinely administered during the study period (Wu et al., 2020).

### 2.4 Observation indicators

#### 2.4.1 Clinical efficacy indicators

According to the international CINV evaluation criteria (Jordan et al., 2025), the following indicators were recorded during the 0–120 h period after chemotherapy: (1) Complete response rate (CR): the proportion of patients with no vomiting, no significant nausea, and no need for rescue antiemetic therapy; (2) No nausea rate (NCR): the proportion of patients without nausea during 0–120 h after chemotherapy; (3) No vomiting rate (NVR): the proportion of patients without vomiting during 0–120 h after chemotherapy; (4) No rescue treatment rate (NRR): the proportion of patients who did not require additional antiemetic drugs after chemotherapy. The occurrences of acute CINV (0–24 h) and delayed CINV (24–120 h) were also recorded.

#### 2.4.2 Quality of life indicators

Quality of life was assessed at baseline (upon enrollment) and after chemotherapy using the European Organization for Research and Treatment of Cancer Quality of Life Questionnaire (EORTC QLQ-C30) (Cocks et al., 2023). This questionnaire contains 30 items, including functional scales (physical, emotional, cognitive, social, etc.), symptom scales (nausea and vomiting, pain, fatigue, etc.), and global health status. The score ranges from 0 to 100 points. Higher scores in the functional scales and global health status indicate better quality of life, while higher scores in the symptom scales indicate a heavier symptom burden.

#### 2.4.3 Safety indicators

Adverse reactions occurring during the study period were recorded, including headache, fatigue, constipation, infusion reactions, and other drug-related adverse events. The time of occurrence, severity, and management measures were also documented.

### 2.5 Cost data collection

This study mainly conducted pharmacoeconomic evaluation from the perspective of the healthcare system while also considering patients’ economic burden. Direct medical costs incurred during chemotherapy were collected, including: (1) Drug costs: including antiemetic drugs, chemotherapy drugs, and adjuvant medications; (2) Examination costs: including routine blood tests, liver and renal function tests, electrolyte tests, and imaging examinations; (3) Treatment costs: including infusion, blood transfusion, and other treatment-related costs; (4) Hospitalization costs: including treatment-related and supportive medical service costs incurred during hospitalization. Drug prices were based on the actual procurement prices of the hospital. Aprepitant capsules were supplied as a 3-day treatment package (125 mg on Day 1 followed by 80 mg on Days 2 and 3), with a procurement cost of 445 RMB per treatment course. Aprepitant injection was administered as a single intravenous dose of 130 mg on Day 1, with a procurement cost of 139.13 RMB per treatment course. Therefore, each chemotherapy cycle required one complete treatment course of either formulation. All costs were calculated in Chinese Yuan (RMB).

### 2.6 Pharmacoeconomic evaluation methods

(1) Cost-effectiveness analysis (CEA): Clinical efficacy indicator (CR rate) was used as the effectiveness outcome to calculate the cost-effectiveness ratio and the incremental cost-effectiveness ratio (ICER): ICER = (C1 _ C0)/(E1 _ E0). Where C1 and E1 represent the cost and effectiveness of the injection group, and C0 and E0 represent the cost and effectiveness of the capsule group. (2) Cost-utility analysis (CUA): Changes in EORTC QLQ-C30 global health status scores were used as surrogate utility outcomes to calculate the incremental cost-utility ratio (ICUR) and compare the relative economic value of the two treatment strategies. Because preference-based utility values and QALY conversion were not available, the resulting ICUR values should be interpreted as exploratory indicators rather than conventional cost-utility estimates. (3) Uncertainty analysis: Probabilistic sensitivity analysis was conducted using Monte Carlo simulation (10,000 iterations) implemented in R software (version 4.3.1). Cost parameters were assumed to follow a gamma distribution because costs are non-negative and right-skewed, whereas effectiveness parameters (CR rate and quality-of-life improvement) were modeled using beta distributions. Incremental costs and incremental effectiveness were simultaneously sampled to generate cost-effectiveness planes and cost-effectiveness acceptability curves (CEACs). The CEACs illustrate the probability that aprepitant injection is considered cost-effective across a range of willingness-to-pay (WTP) thresholds. For the two-strategy comparison, the probability of the capsule strategy being cost-effective was calculated as the complement of the probability of the injection strategy being cost-effective at each willingness-to-pay threshold.

### 2.7 Propensity score matching

To reduce selection bias and confounding caused by non-randomized treatment allocation in this real-world observational study, PSM was performed to improve baseline comparability between the aprepitant injection group and the aprepitant capsule group. Propensity scores were estimated using a multivariable logistic regression model, with treatment group assignment as the dependent variable. Covariates included in the matching model were selected based on their potential influence on antiemetic efficacy and clinical outcomes, including age, sex, BMI, tumor type, chemotherapy emetogenicity (moderately versus highly emetogenic), chemotherapy cycle, ECOG performance status, prior history of CINV, and concomitant non-antiemetic medication use. Antiemetic agents with independent antiemetic activity, such as olanzapine or additional dexamethasone beyond the predefined prophylactic regimen, were not routinely used and therefore were not included as matching covariates. A 1:1 nearest-neighbor matching algorithm without replacement was applied to minimize baseline imbalance between groups. After matching, 60 matched pairs were successfully generated from the original cohort, resulting in 60 patients in the injection group and 60 patients in the capsule group for final comparative analysis. Baseline demographic and clinical characteristics were compared before and after matching to assess matching effectiveness. Before matching, there was substantial imbalance in sample size between the two groups, although major clinical variables were generally comparable. After PSM, no statistically significant differences were observed between the two groups in major baseline variables (all P > 0.05), indicating satisfactory balance and improved intergroup comparability (Table 1). By reducing measurable confounding factors, PSM enhanced the internal validity and robustness of subsequent analyses of clinical efficacy, safety, quality of life, and pharmacoeconomic outcomes.

**TABLE 1:** Comparison of baseline characteristics before and after propensity score matching between the two groups.

Before PSM	Injection group (n=306)	Capsule group (n=62)	*t/x2*	*P*
Male	161 (52.61)	31 (50.00)	0.142	0.706
Female	145 (47.39)	31 (50.00)	-	-
Age (years)	57.42 ± 10.18	55.96 ± 9.84	1.026	0.306
BMI (kg/m2)	23.71 ± 3.42	23.38 ± 3.19	0.694	0.488
Gastrointestinal tumors	l09 (35.62)	23 (37. L0)	0.058	0.8L0
Lung cancer	88 (28.76)	l8 (29.03)	0.002	0.967
Breast cancer	67 (2L.90)	l3 (20.97)	0.026	0.872
Other tumors	42 (l3.73)	8 (l2.90)	0.029	0.865
Highly emetogenic chemotherapy	l96 (64.05)	39 (62.90)	0.030	0.863
Moderately emetogenic chemotherapy	ll0 (35.95)	23 (37. L0)	-	-
First chemotherapy cycle	l74 (56.86)	33 (53.23)	0.27L	0.603
ECOG score ≥2	82 (26.80)	l8 (29.03)	0. L29	0.7L9
Prior CINV history	9L (29.74)	2L (33.87)	0.4L8	0.5L8
After PSM	-	-	-	-
Male	3L (5L.67)	30 (50.00)	0.033	0.856
Female	29 (48.33)	30 (50.00)	-	-
Age (years)	56.2L ± 9.74	55.87 ± 9.68	0. L92	0.848
BMI (kg/m2)	23.46 ± 3.25	23.39 ± 3. L7	0. Ll8	0.906
Gastrointestinal tumors	22 (36.67)	23 (38.33)	0.036	0.849
Lung cancer	l7 (28.33)	l8 (30.00)	0.040	0.842
Breast cancer	l3 (2L.67)	l2 (20.00)	0.050	0.823
Other tumors	8 (l3.33)	7 (ll.67)	0.078	0.780
Highly emetogenic chemotherapy	38 (63.33)	39 (65.00)	0.038	0.845
Moderately emetogenic chemotherapy	22 (36.67)	2L (35.00)	-	-
First chemotherapy cycle	33 (55.00)	32 (53.33)	0.033	0.856
ECOG score ≥2	l7 (28.33)	l8 (30.00)	0.040	0.842
Prior CINV history	l9 (3L.67)	20 (33.33)	0.038	0.845

### 2.8 Statistical methods

Propensity score matching (PSM) and pharmacoeconomic modeling, including Monte Carlo simulation and cost-effectiveness acceptability curve (CEAC) generation, were performed using R software (version 4.3.1; R Foundation for Statistical Computing, Vienna, Austria). Conventional statistical analyses and graphical presentations were conducted using GraphPad Prism 9.0 (GraphPad Software, San Diego, CA, USA). Ontinuous variables were first tested for normality using the Shapiro–Wilk test. Variables conforming to a normal distribution were expressed as (^*x*^±^s^), and comparisons between groups were performed using the independent samples t-test. Variables not conforming to a normal distribution were expressed as median (interquartile range), and comparisons between groups were performed using non-parametric tests. Categorical data were expressed as number of cases and percentages, and comparisons between groups were performed using the χ2 test. A P value <0.05 was considered statistically significant.

## 3 Results

### 3.1 Comparison of baseline characteristics between the two groups

A total of 368 patients with solid tumors receiving moderately to highly emetogenic chemotherapy were initially screened, including 306 patients in the aprepitant injection group and 62 patients in the aprepitant capsule group. To minimize selection bias and improve comparability, 1:1 PSM was performed, resulting in 60 matched pairs for final analysis. Baseline demographic and clinical characteristics before and after PSM are presented in Table 1. After matching, no statistically significant differences were observed between the two groups in major baseline variables, including age, sex, BMI, tumor type, chemotherapy emetogenicity, chemotherapy cycle, ECOG performance status, and prior CINV history (all P > 0.05), indicating satisfactory baseline balance and improved comparability. These results suggest that PSM effectively reduced measurable baseline confounding and provided a reliable foundation for subsequent comparative analyses of clinical efficacy, quality of life, safety, and pharmacoeconomic outcomes.

### 3.2 Comparison of clinical efficacy between the two groups

During the overall 0–120 h observation period following chemotherapy, both the aprepitant injection group and the aprepitant capsule group demonstrated favorable control of CINV. After propensity score matching, the overall CR rate was 78.33% in the injection group and 76.67% in the capsule group, with no statistically significant difference between groups (P > 0.05). Further subgroup analysis showed that the acute-phase (0–24 h) CR rates were 86.67% and 85.00% in the injection and capsule groups, respectively, while delayed-phase (24–120 h) CR rates were 75.00% and 73.33%, respectively. No statistically significant intergroup differences were identified at either stage (all P > 0.05). Similarly, no significant differences were observed between the two groups in NCR, NVR, or NRR (all P > 0.05). Although the injection group showed numerically slightly higher efficacy across most indicators, the differences were not statistically significant, indicating that no statistically significant differences in antiemetic efficacy were observed between the two formulations under standard triple antiemetic prophylaxis. Overall, no statistically significant differences were observed between the two formulations in preventing acute or delayed CINV. However, the present study was not specifically designed or powered to formally demonstrate equivalence or non-inferiority between treatment groups. Detailed efficacy outcomes are presented in Table 2.

**TABLE 2:** Comparison ofCINV Control Between the Two Groups.

Outcome	Injection group (n=60)	Capsule group (n=60)	*x* ^2^	*P*
Overall CR rate (0–120 h)	47 (78.33%)	46 (76.67%)	0.048	0.827
Acute phase CR rate (0–24 h)	52 (86.67%)	51 (85.00%)	0.069	0.793
Delayed phase CR rate (24–120 h)	45 (75.00%)	44 (73.33%)	0.045	0.832
NCR	43 (71.67%)	42 (70.00%)	0.041	0.840
NVR	49 (81.67%)	48 (80.00%)	0.053	0.818
NRR	51 (85.00%)	50 (83.33%)	0.064	0.800

### 3.3 Comparison of quality of life between the two groups

**FIGURE 2:**
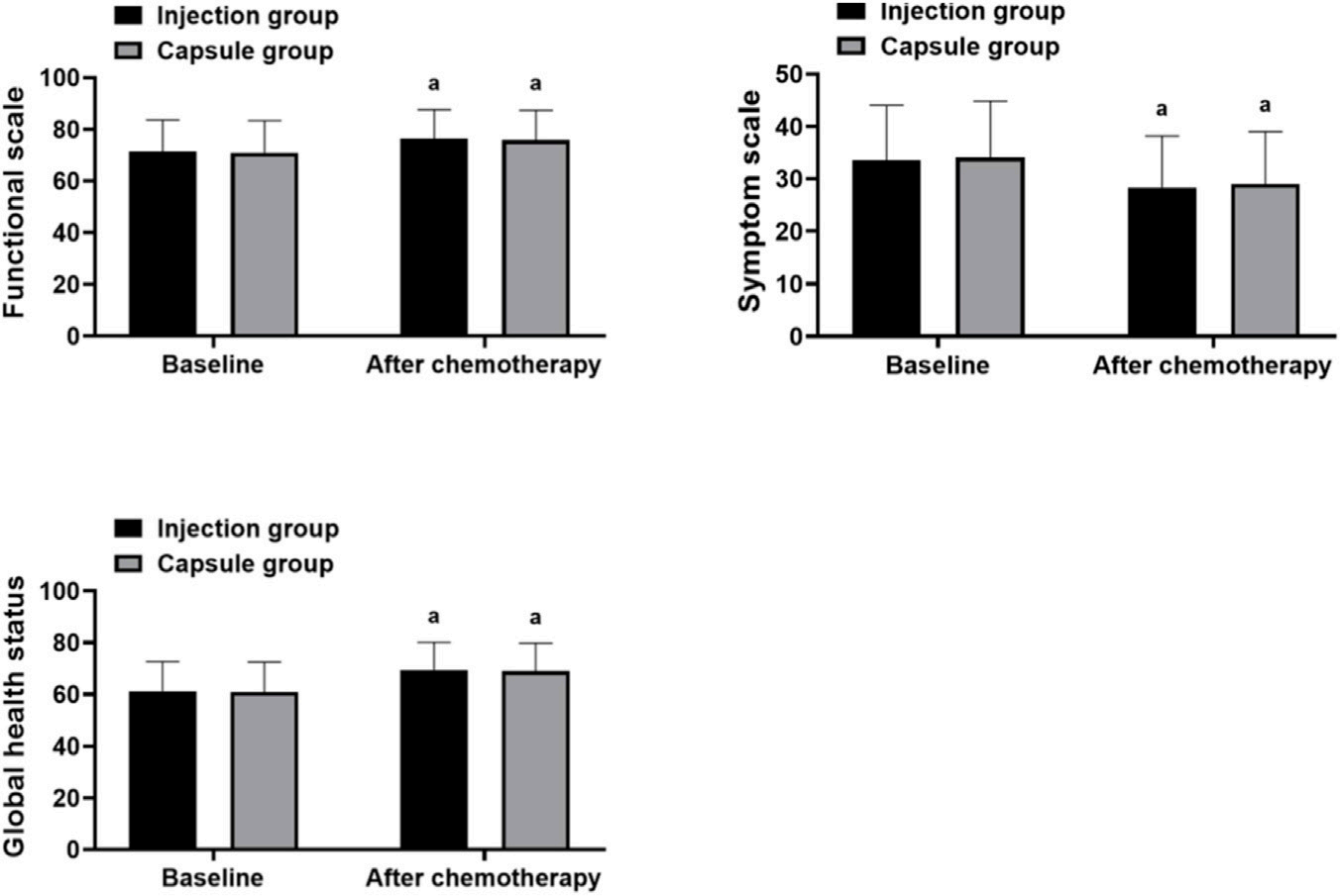
Comparison of EORTC QLQ-C30 quality of life scores between the two groups (points) Note: a indicates P < 0.05 compared with baseline in the same group.

The EORTC QLQ-C30 questionnaire was used to evaluate patients’ quality of life. The results showed that after chemotherapy, the global health status scores and functional scale scores of both groups increased compared with baseline, while the symptom scale scores decreased compared with baseline, indicating that antiemetic therapy could improve patients’ quality of life to a certain extent. The comparison between groups showed no statistically significant differences in quality-of-life scores at baseline or after chemotherapy between the injection group and the capsule group (P > 0.05), suggesting that the two antiemetic regimens had comparable effects in improving patients’ quality of life. The specific results are shown in Table 3 and Figure 2.

**TABLE 3:** Comparison of EORTC QLQ-C30 quality of life scores between the two groups (points).

Domain	Injection group (n=60)	Capsule group (n=60)	*t*	*P*
Functional scale	-	-	-	-
Baseline	71.42 ± 12.18	70.96 ± 12.44	0.264	0.791
After chemotherapy	76.35 ± 11.27^a^	75.81 ± 11.53^a^	0.334	0.738
Symptom scale	-	-	-	-
Baseline	33.58 ± 10.46	34.12 ± 10.71	0.360	0.718
After chemotherapy	28.37 ± 9.82^a^	29.04 ± 9.96^a^	0.479	0.632
Global health status	-	-	-	-
Baseline	61.28 ± 11.34	60.84 ± 11.58	0.271	0.786
After chemotherapy	69.42 ± 10.73^a^	68.97 ± 10.81^a^	0.295	0.768

a indicates P < 0.05 compared with baseline in the same group.

### 3.4 Comparison of safety outcomes

Treatment-related adverse events were recorded throughout the study period. The most commonly observed adverse events included headache, fatigue, constipation, and infusion-related reactions. No serious adverse events related to antiemetic therapy were observed in either group. The incidence of individual adverse events and the overall adverse event rate did not differ significantly between the injection and capsule groups (all P > 0.05), indicating comparable safety profiles between the two formulations. Detailed safety outcomes are presented in Table 4.

**TABLE 4:** Comparison of safety outcomes.

Adverse event	Injection group (n=60)	Capsule group (n=60)	*x* ^2^	*P*
Headache	4 (6.67%)	5 (8.33%)	0.124	0.725
Fatigue	6 (10.00%)	7 (11.67%)	0.089	0.765
Constipation	5 (8.33%)	6 (10.00%)	0.100	0.752
Infusion reaction	2 (3.33%)	0 (0.00%)	2.034	0.154
Total adverse events	17 (28.33%)	18 (30.00%)	0.041	0.840

### 3.5 Comparison of medical costs between the two groups

**FIGURE 3:**
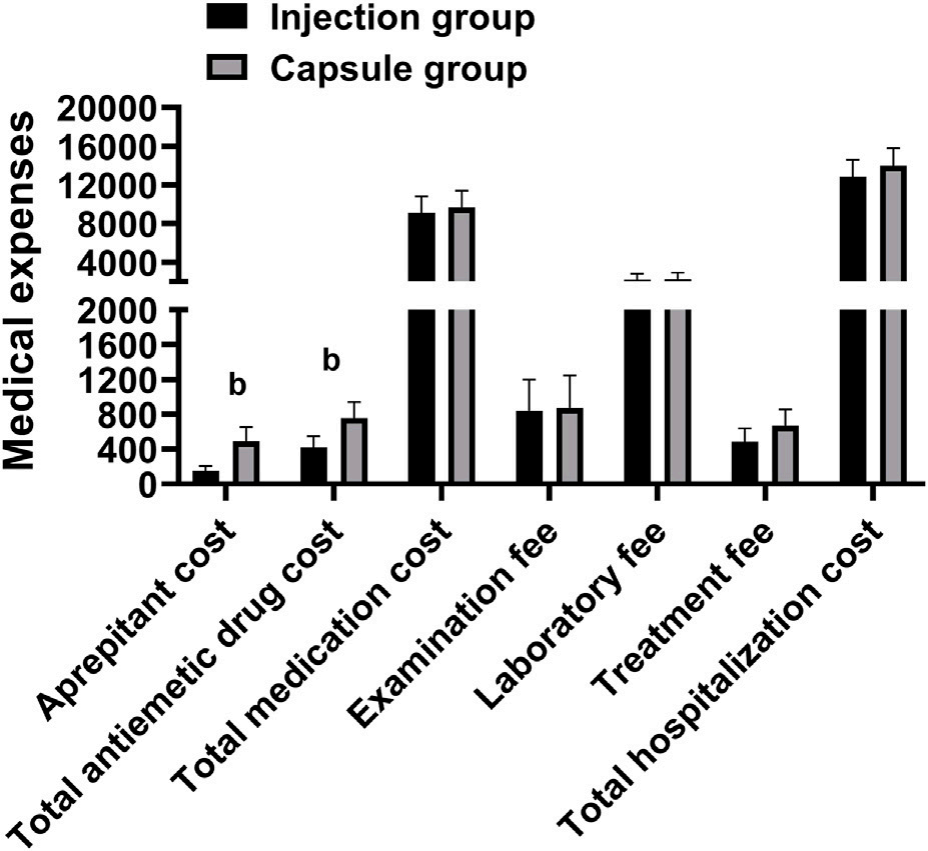
Comparison of medical costs between the two groups (RMB). Note: b indicates P < 0.05 between groups.

In this study, medical costs were analyzed according to the actual cost structure during hospitalization. Aprepitant acquisition cost was calculated separately, while total antiemetic drug cost included aprepitant, ondansetron, and dexamethasone. Total medication cost included antiemetic drugs, chemotherapy drugs, and adjuvant medications. Total hospitalization cost was calculated as the sum of total medication cost, examination fees, laboratory fees, and treatment fees. The results showed that the aprepitant cost in the injection group was significantly lower than that in the capsule group (153.04 ± 54.71 RMB vs. 489.50 ± 164.83 RMB, P < 0.001). The total antiemetic drug cost was also significantly lower in the injection group than in the capsule group (421.36 ± 128.47 RMB vs. 756.42 ± 186.35 RMB, P < 0.001). There were no statistically significant differences between the two groups in examination fees, laboratory fees, or treatment fees (all P > 0.05), indicating that the difference in hospitalization cost was mainly attributable to the difference in antiemetic drug acquisition cost. In terms of total medication cost, the injection group showed a lower average cost than the capsule group (9,125.64 ± 1,684.37 RMB vs. 9,786.53 ± 1792.46 RMB, P > 0.05). The total hospitalization cost in the injection group was also lower than that in the capsule group (12,856.42 ± 1745.38 RMB vs. 13,968.57 ± 1832.64 RMB), with a statistically significant difference (P < 0.05). These findings suggest that aprepitant injection may reduce the overall economic burden while maintaining comparable clinical efficacy. Detailed cost comparisons are shown in Table 5 and Figure 3.

**TABLE 5:** Comparison of medical costs between the two groups (RMB).

Cost item	Injection group (n=60)	Capsule group (n=60)	*t*	*P*
Aprepitant cost	153.04 ± 54.71	489.50 ± 164.83	15.024	<0.001
Total antiemetic drug cost	421.36 ± 128.47	756.42 ± 186.35	11.467	<0.001
Total medication cost	9,125.64 ± 1,684.37	9,786.53 ± 1792.46	1.945	0.054
Examination fee	842.53 ± 356.72	875.41 ± 372.68	0.494	0.622
Laboratory fee	2,186.47 ± 648.35	2,245.63 ± 672.54	0.490	0.625
Treatment fee	486.35 ± 152.74	672.48 ± 183.56	1.865	0.064
Total hospitalization cost	12,856.42 ± 1745.38	13,968.57 ± 1832.64	2.137	0.035

### 3.6 Pharmacoeconomic evaluation

Based on the direct medical cost analysis and clinical efficacy outcomes, CEA was performed to further compare the pharmacoeconomic value of aprepitant injection and aprepitant capsules. The overall CR rate during the 0–120 h observation period was used as the primary effectiveness indicator. The average total hospitalization cost was 14,856.42 RMB in the injection group and 13,968.57 RMB in the capsule group, resulting in an incremental cost difference of −1,112.15 RMB. The CR rate was 78.33% in the injection group and 76.67% in the capsule group, corresponding to an incremental effectiveness gain of 1.66%.

According to the standard ICER formula:

$$\text{ICER}=\left(\text{Cost}\_\text{injection }\_\text{ Cost}\_\text{capsule}\right)/\left(\text{Effect}\_\text{injection }\_\text{ Effect}\_\text{capsule}\right)$$

$$\text{Thus}:\text{ICER}=\left(-1112.15\right)\;/\;0.0166\approx -66,996.99\text{ RMB}$$

The ICER was −66,996.99 RMB per additional complete response, reflecting a reduction in total cost for a slight improvement in efficacy. This indicates that aprepitant injection is a dominant strategy in pharmacoeconomic terms. However, given that no statistically significant differences were observed in clinical efficacy between the two formulations, this conclusion reflects economic advantage rather than a clinically significant difference. This finding demonstrates a clear pharmacoeconomic advantage of the injection regimen. The specific results are shown in Table 6.

**TABLE 6:** Pharmacoeconomic evaluation results.

Indicator	Injection group (n=60)	Capsule group (n=60)	Incremental value
Average cost (RMB)	14,856.42	13,968.57	−112.15
CR rate (%)	78.33	76.67	1.66
ICER (RMB)	-	-	−66,996.99

### 3.7 Cost-utility analysis

CUA was performed using the change in EORTC QLQ-C30 global health status score as a surrogate utility indicator. Because preference-based utility measures were not available, the analysis was intended to provide an exploratory assessment of costs relative to quality-of-life improvement rather than a formal QALY-based cost-utility evaluation. In the injection group, the global health status score increased from 61.28 ± 11.34 at baseline to 69.42 ± 10.73 after chemotherapy, corresponding to a utility gain (Δ U) of 8.14 points. In the capsule group, the score increased from 60.84 ± 11.58 to 68.97 ± 10.81, corresponding to a utility gain of 8.13 points. Therefore, the incremental utility gain between groups was 0.01 points.

Using the ICUR formula:

$$\text{ICUR}=\left(\text{Cost}\_\text{injection }\_\text{ Cost}\_\text{capsule}\right)/\left(\text{Utility}\_\text{injection }\_\text{ Utility}\_\text{capsule}\right)$$

$$\text{Thus}:\text{ICUR}=\left(-1112.15\right)/0.01=-111,215.00\text{ RMB}/\text{point}$$

The negative ICUR indicates that aprepitant injection achieved comparable quality-of-life improvement while reducing overall hospitalization costs, further supporting its economic advantage from the utility perspective. The specific results are shown in Table 7.

**TABLE 7:** Cost-utility analysis results.

Indicator	Injection group (n=60)	Capsule group (n=60)	Incremental value
Average cost (RMB)	12,856.42	13,968.57	_−_1,112.15
Baseline global health score (points)	61.28	60.84	-
Post-chemotherapy global health score (points)	69.42	68.97	-
Utility increment (Δu, points)	8.14	8.13	0.01
ICUR (RMB/point)	-	-	−111,215.00

### 3.8 Uncertainty analysis

**FIGURE 4:**
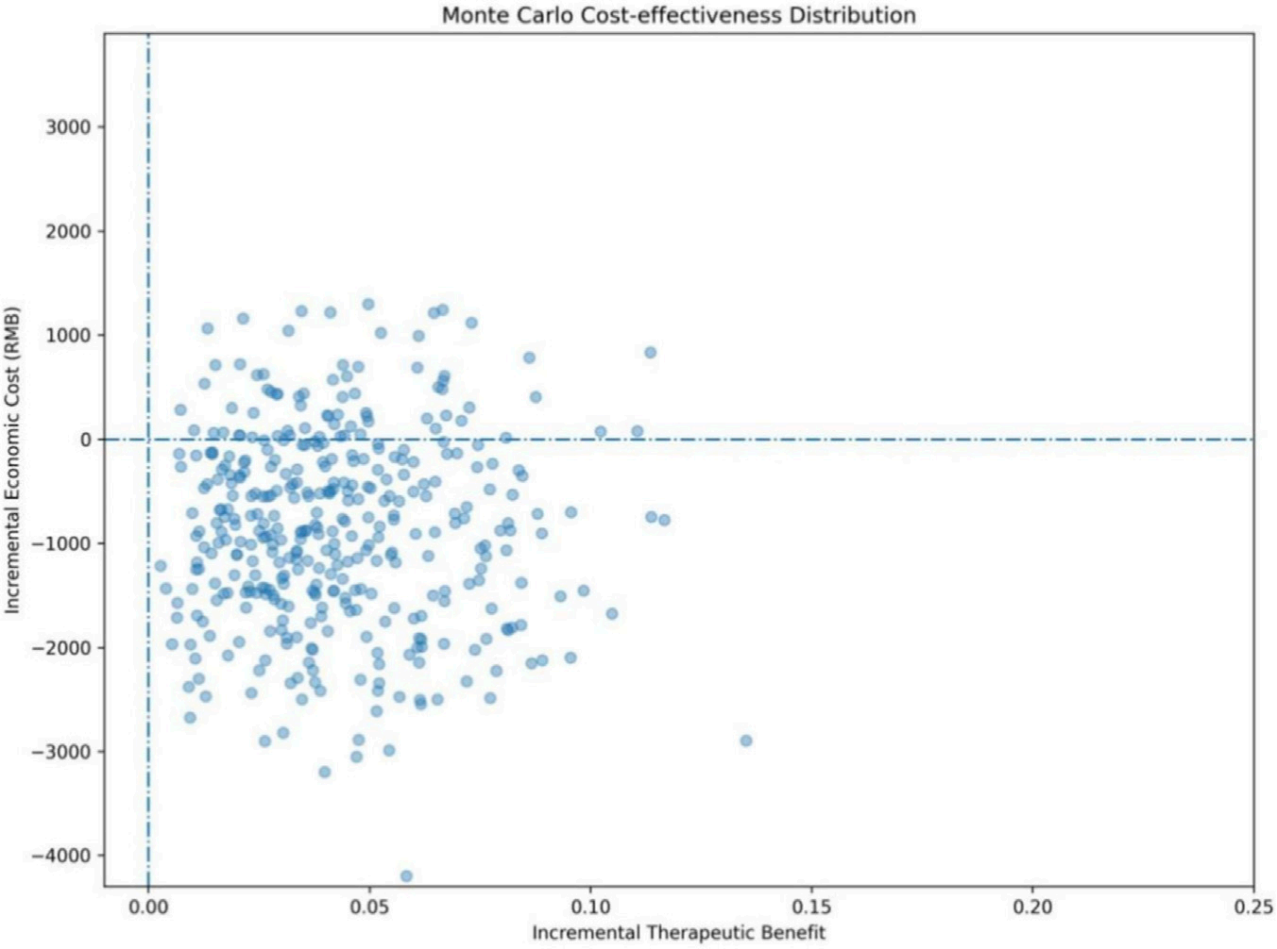
Cost-effectiveness plane comparing aprepitant injection and aprepitant capsules. Note: The x-axis represents incremental effectiveness (difference in complete response rate), and the y-axis represents incremental cost (difference in total hospitalization cost, RMB).

**FIGURE 5:**
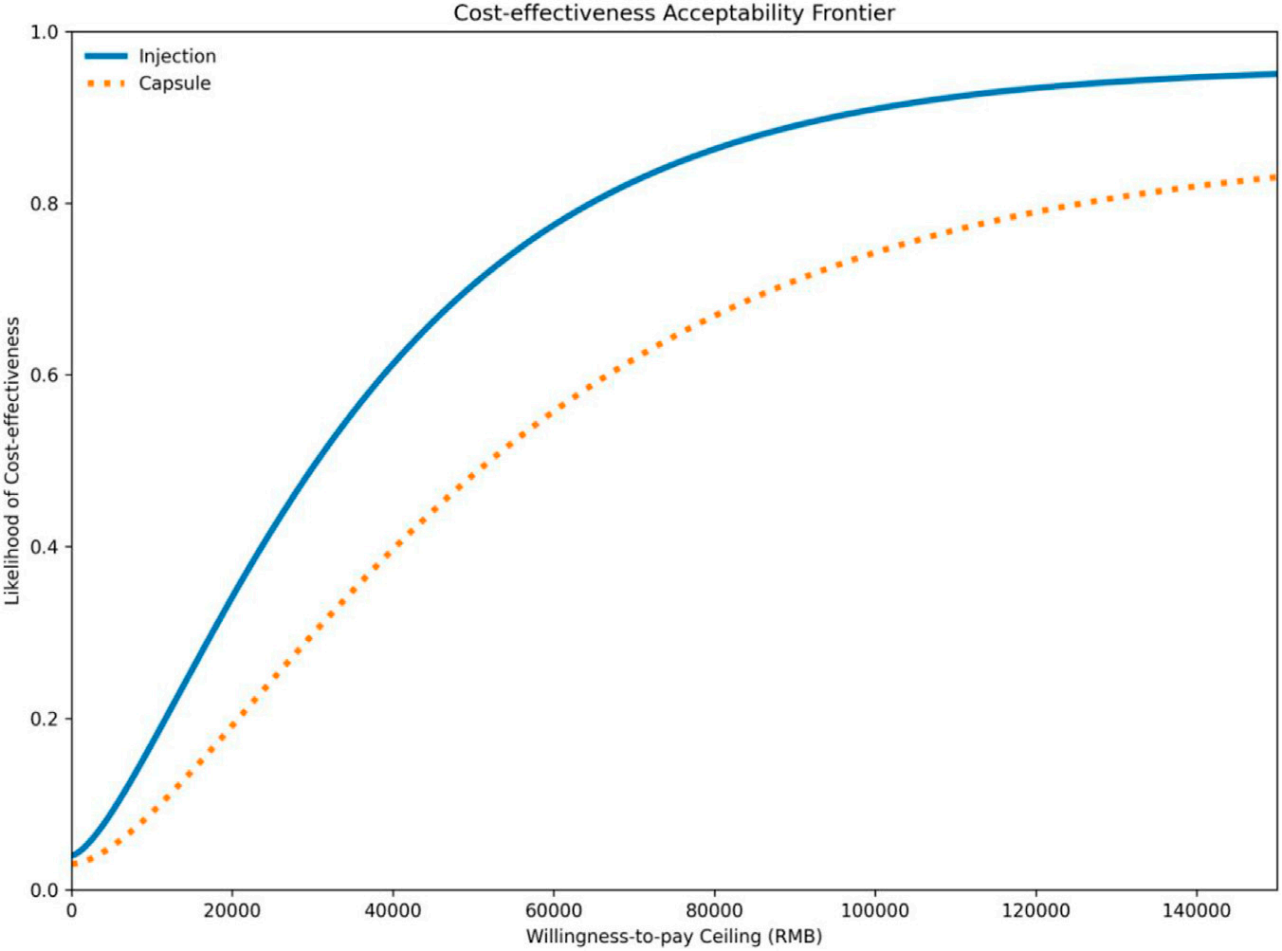
Cost-effectiveness acceptability curve comparing aprepitant injection and aprepitant capsules. Note: The x-axis represents the willingness-to-pay threshold (RMB per additional complete response achieved), and the y-axis represents the probability of being cost-effective.

In the uncertainty analysis, Monte Carlo simulation was used to perform probabilistic sensitivity analysis on cost and effectiveness parameters. The simulation results were presented in a cost-effectiveness plane, as shown in Figure 4. In the cost-effectiveness plane, the x-axis represents incremental effectiveness (difference in CR rate between the injection and capsule groups), while the y-axis represents incremental cost (difference in total hospitalization cost between groups). The results showed that most simulated points were distributed in the cost-saving region, indicating that in most scenarios the aprepitant injection regimen could reduce medical costs while achieving comparable therapeutic effects, demonstrating favorable economic advantages. Figure 5 presents the cost-effectiveness acceptability curve across a range of willingness-to-pay thresholds. As the willingness-to-pay threshold increased, the probability of aprepitant injection being considered cost-effective increased progressively, whereas the corresponding probability for aprepitant capsules decreased proportionally. The two probabilities were complementary at each threshold, reflecting the two-strategy comparison framework.

## 4 Discussion

CINV remains one of the most common and clinically significant adverse effects associated with antitumor chemotherapy, particularly in patients receiving moderately and highly emetogenic regimens. Despite major advances in antiemetic prophylaxis, delayed-phase CINV continues to represent an important unmet supportive care challenge in real-world oncology practice (Pat et al., 2022), (Navari et al., 2023). Poorly controlled CINV not only causes substantial physical discomfort, nutritional compromise, dehydration, and psychological distress, but may also negatively influence chemotherapy adherence, thereby potentially affecting long-term treatment continuity and therapeutic outcomes (Schwartzberg et al., 2023)- (Navari and Bonizzoni, 2025; Belluomini et al., 2024). Therefore, modern antiemetic strategy evaluation should extend beyond efficacy alone and comprehensively consider quality-of-life outcomes, treatment convenience, and healthcare resource utilization. Against this background, the present real-world study compared aprepitant injection and aprepitant capsules in patients receiving moderately to highly emetogenic chemotherapy and demonstrated that while no statistically significant differences in antiemetic efficacy or quality-of-life outcomes were observed between the two formulations, aprepitant injection showed superior pharmacoeconomic performance due primarily to lower drug acquisition costs and reduced total hospitalization expenditures.

The clinical efficacy findings of this study demonstrated that both formulations of aprepitant provided favorable control of acute and delayed CINV under guideline-recommended triple antiemetic prophylaxis. No statistically significant differences were observed between the injection and capsule groups in overall CR rate, acute-phase CR rate, delayed-phase CR rate, NCR, NVR, or NRR. These results suggest that no statistically significant differences in antiemetic outcomes were detected between the two formulations in the present study. Mechanistically, aprepitant exerts antiemetic effects through selective blockade of substance P-mediated NK- one receptor pathways, thereby complementing 5-HT3 receptor antagonists and dexamethasone in both acute and delayed phases (Zhu et al., 2024). Our findings are generally consistent with previous clinical studies and antiemetic guideline recommendations supporting NK- one receptor antagonist use in moderately and highly emetogenic chemotherapy (Okuyama et al., 2025; Filetti et al., 2023).

Although efficacy differences were not statistically significant, the injection group consistently demonstrated numerically slightly higher CR-related indicators. This trend may have practical clinical implications. Intravenous administration ensures accurate pre-chemotherapy delivery under medical supervision, potentially improving adherence and reducing missed or delayed dosing compared with multi-day oral regimens (Horii et al., 2024). In addition, injectable formulations may be particularly advantageous in patients with baseline nausea, swallowing difficulty, gastrointestinal dysfunction, or poor compliance. Since delayed CINV prevention with oral capsules requires continued administration over multiple days, adherence-related variability may influence real-world effectiveness, whereas the single-dose injectable strategy may offer more predictable prophylactic coverage (Gao et al., 2023). Therefore, although no statistically significant differences in antiemetic outcomes were observed between the two formulations, the present study was not powered to formally establish efficacy equivalence. Nevertheless, practical administration advantages may still support the clinical utility of the injectable formulation in selected patient populations.

Quality-of-life analysis further demonstrated that both regimens significantly improved functional status and global health scores while reducing symptom burden following chemotherapy. This finding underscores the importance of effective antiemetic prophylaxis as a key component of supportive oncology care. By reducing nausea, vomiting, appetite loss, fatigue, and associated distress, both aprepitant formulations contributed to improved patient-reported outcomes. Importantly, the comparable quality-of-life improvements between groups indicate that formulation differences did not substantially alter supportive efficacy from the patient perspective. These findings reinforce that both regimens are clinically effective supportive options, while formulation choice may depend more heavily on economic and practical considerations than on symptomatic efficacy alone. In terms of safety, both formulations were generally well tolerated. No serious treatment-related adverse events were observed during the study period, and the incidences of common adverse events, including headache, fatigue, constipation, and infusion-related reactions, were comparable between groups. These findings suggest that the two formulations have similar safety profiles when used as part of standard triple antiemetic prophylaxis (Johns and Lawrence, 2024).

The most clinically meaningful distinction between the two regimens emerged from the pharmacoeconomic analysis. This study demonstrated that aprepitant injection significantly reduced both direct antiemetic drug costs and total hospitalization costs compared with aprepitant capsules while no statistically significant differences in efficacy or quality-of-life outcomes were observed between groups. The economic advantage was primarily attributable to differences in drug acquisition costs under the institutional procurement system. Specifically, one chemotherapy cycle required a complete 3-day oral aprepitant regimen (125 mg on Day 1 followed by 80 mg on Days 2 and 3), corresponding to a procurement cost of 445 RMB per treatment course, whereas aprepitant injection was administered as a single 130 mg intravenous dose at a cost of 139.13 RMB per course. Given that no statistically significant differences in antiemetic efficacy were observed between the two formulations, the substantially lower acquisition cost of the injectable formulation translated directly into lower antiemetic drug expenditure and ultimately reduced overall hospitalization costs. Since examination fees, laboratory fees, and treatment fees were generally similar between groups, the observed cost savings were mainly driven by medication-related expenditures rather than broader differences in healthcare utilization. For patients undergoing multiple chemotherapy cycles, these per-cycle savings may accumulate over time and potentially reduce the long-term economic burden on both patients and healthcare systems. Cost-effectiveness analysis further confirmed this economic advantage. The negative ICER observed in this study indicates that aprepitant injection represented a dominant pharmacoeconomic strategy, meaning that it reduced total cost while no statistically significant differences in therapeutic efficacy were observed between groups (Henson et al., 2020). Similarly, cost-utility analysis demonstrated a negative ICUR, suggesting lower cost relative to the observed improvement in quality-of-life scores. However, because EORTC QLQ-C30 score changes rather than preference-based utility values were used, the ICUR should be interpreted cautiously and cannot be directly compared with conventional willingness-to-pay thresholds. Together, these findings support the conclusion that aprepitant injection offers superior value-based care under current pricing conditions. Although this study used EORTC QLQ-C30 global health score rather than formal QALY conversion for utility analysis, the approach remains practical and clinically relevant within the context of supportive care and short-term symptom management (Milazzo et al., 2020; Stone et al., 2023).

Probabilistic sensitivity analysis using Monte Carlo simulation and cost-effectiveness acceptability curves further strengthened the robustness of these conclusions. Most simulated scenarios favored the injection strategy within cost-saving regions, and CEAC analysis demonstrated that the probability of aprepitant injection being considered cost-effective increased steadily as willingness-to-pay thresholds increased. Across a broad range of thresholds, aprepitant injection maintained a higher probability of being cost-effective than aprepitant capsules, supporting the robustness of the pharmacoeconomic findings. These findings suggest that the pharmacoeconomic advantages of aprepitant injection were not solely dependent on point estimates but remained relatively stable under plausible variations in cost and efficacy assumptions.

Several limitations should be acknowledged. First, this was a single-center retrospective real-world study with limited external generalizability. Second, although propensity score matching reduced measurable baseline confounding and improved comparability between groups, residual confounding from unmeasured variables cannot be completely excluded. In addition, the relatively limited sample size after matching may have reduced the ability to detect small differences in clinical outcomes between the two formulations. Third, only direct medical costs were analyzed, while indirect societal costs were not included. Fourth, the utility assessment was based on changes in EORTC QLQ-C30 scores rather than preference-based utility measures. Consequently, QALY conversion was not performed, and the resulting ICUR values cannot be directly interpreted against conventional willingness-to-pay thresholds. Future multicenter studies incorporating larger populations, broader economic perspectives, and standardized QALY-based analyses are warranted to further validate these findings.

## 5 Conclusion

Aprepitant injection and aprepitant capsules showed no statistically significant differences in clinical efficacy, safety, or quality-of-life outcomes in this matched retrospective cohort. However, aprepitant injection provided superior pharmacoeconomic advantages through lower antiemetic acquisition costs, reduced total hospitalization expenditures, and more favorable cost-effectiveness and cost-utility profiles. Under current real-world clinical and pricing conditions, aprepitant injection may represent a cost-saving supportive care alternative for appropriate patients undergoing emetogenic chemotherapy. These findings provide practical evidence for individualized antiemetic regimen optimization and healthcare resource allocation, while larger prospective multicenter pharmacoeconomic studies remain necessary for further validation.

## Data Availability

The original contributions presented in the study are included in the article/supplementary material, further inquiries can be directed to the corresponding authors.
